# Do nonsteroidal anti-inflammatory drugs increase the risk of atrial fibrillation after non-cardiac surgery? Insights from translational and clinical research

**DOI:** 10.1186/s44158-025-00303-5

**Published:** 2025-11-19

**Authors:** Eleni Laou, Styliani Mitta, Eleni Voutsadaki, Nicoleta Ntalarizou, Paraskevi Tselioti, Andrea Cortegiani, Athanasios Chalkias

**Affiliations:** 1 Department of Anesthesiology, Agia Sophia Children’s Hospital, Athens, Greece; 2 Department of Anesthesiology, General Hospital of Piraeus “Tzaneio” , Piraeus, Greece; 3 Department of Critical Care Medicine, General Hospital of Piraeus “Tzaneio” , Piraeus, Greece; 4 Department of Anesthesia, Analgesia, Intensive Care and Emergency, University Hospital Policlinico Paolo Giaccone, Palermo, Italy; 5 Department of Precision Medicine in Medical, Surgical and Critical Care (Me.Pre.C.C.), University of Palermo, Palermo, Italy; 6 Institute for Translational Medicine and Therapeutics, University of Pennsylvania Perelman School of Medicine, Philadelphia, PA USA; 7 OUTCOMES RESEARCH Consortium®, Houston, TX USA

**Keywords:** Non-cardiac surgery, Non-steroidal anti-inflammatory drugs, Perioperative, Atrial fibrillation, Translational research, Anesthesiology, Perioperative medicine, Critical care

## Abstract

Non-steroidal anti-inflammatory drugs (NSAIDs) are integral to multimodal analgesic strategies after non-cardiac surgery, aimed at minimizing opioid exposure. Although their analgesic and anti-inflammatory efficacy is well established, emerging evidence raises concerns that perioperative NSAID use may increase the risk of postoperative atrial fibrillation (POAF). Mechanistic studies suggest multiple pathways for this association, including cyclooxygenase inhibition, renin–angiotensin–aldosterone system activation, oxidative stress, and autonomic dysregulation. In this context, perioperative clinicians face the challenge of optimizing pain control while mitigating cardiovascular risk. This review synthesizes preclinical, translational, and clinical data to delineate the potential impact of NSAIDs on POAF risk after non-cardiac surgery, providing a framework for evidence-informed perioperative management.

## Background

Postoperative atrial fibrillation (POAF) occurs in approximately 20-40% of cardiac and 10-20% of non-cardiac surgeries, representing the most significant form of secondary atrial fibrillation [1]. While the inflammatory response plays a central role in the multifactorial pathogenesis of POAF [2], its clinical course remains incompletely characterized [3–8]. Nevertheless, emerging evidence suggest that POAF is associated with a substantially increased risk of stroke, myocardial infarction, and all-cause mortality within 30 d following non-cardiac surgery [3].

Perioperative medicine and modern multimodal analgesic techniques frequently incorporate non-steroidal anti-inflammatory medications (NSAIDs) as part of preemptive and preventive strategies [4]. By attenuating the body’s inflammatory response to surgical injury and reducing the release of inflammatory mediators, NSAIDs can decrease opioid requirements and facilitate postoperative recovery. This principle forms the foundation of opioid-free anesthesia (OFA) approaches, which employ combinations of non-opioid agents to achieve effective analgesia [5]. However, recent evidence suggests that NSAID use may be associated with an increased risk of perioperative hemodynamic instability, stroke, prolonged hospitalization, and mortality [1, 9–11]. Furthermore, a recent network meta-analysis reported that although OFA regimens reduce postoperative opioid consumption and nausea, they do not demonstrate superior analgesic efficacy compared with opioid-based anesthesia during the initial 24-h postoperative period [12]. These findings underscore the importance of carefully weighing potential complications when considering NSAID use in perioperative analgesic strategies.

This review synthesizes preclinical, translational, and clinical evidence to evaluate the potential role of NSAIDs in the development of POAF following non-cardiac surgery.

## Mechanisms connecting nonsteroidal anti-inflammatory drugs and atrial fibrillation

The interaction between NSAID use and POAF is multifactorial and remains poorly understood, with several mechanisms believed to be involved.

### Inflammation and oxidative stress

**Fig. 1:**
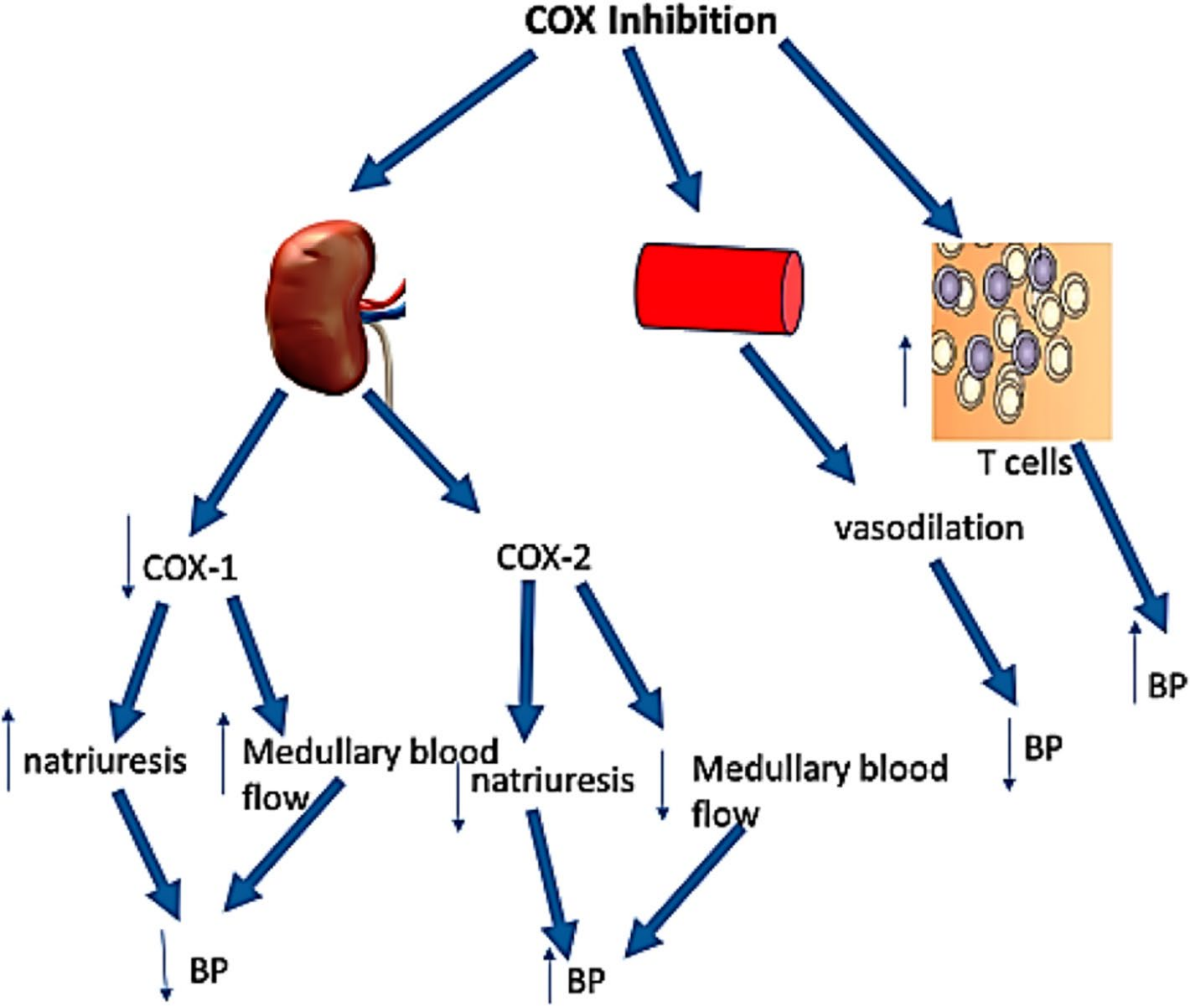
COX inhibition decreases or increases blood pressure through inhibition of renal COX-1 or COX-2, respectively. However, the overwhelming effect of COX inhibition in the vasculature is vasodilatory. Systemic COX inhibition may increase blood pressure through activation of T-cells and promotion of their infiltration into cardiovascular organs. Adapted from reference [17]

Nonsteroidal anti-inflammatory drugs inhibit cyclooxygenase (COX) enzymes, thereby reducing prostaglandin synthesis. Prostaglandins normally play a central role in regulating vascular tone, inflammatory signaling, and renal perfusion. Inhibition of COX-1 provides anti-inflammatory effects, but inhibition of COX-2 disrupts the renin–angiotensin–aldosterone system (RAAS). This disruption promotes sodium and water retention, vasoconstriction, and systemic hypertension, which lead to the release of proinflammatory cytokines and other mediators. In surgical patients, venous congestion and fluid overload stretch blood vessels and organs, causing endothelial activation, oxidative stress, and the release of inflammatory molecules, which perpetuate a damaging cycle of worsening congestion, organ dysfunction, and inflammation [13–15]. These pathophysiological processes increase left atrial pressure and mechanical stretch. Atrial stretch is a well-established driver of structural and electrical remodeling, thereby predisposing the atria to arrhythmogenesis and POAF (Fig. 1) [16, 17].

Furthermore, systemic inflammation and T-cell infiltration into atrial tissue have emerged as critical contributors to the onset of POAF. In patients undergoing elective cardiac surgery, elevated perioperative levels of CD8^+^CD28^null T lymphocytes, a cytotoxic and autoaggressive subset, are independently associated with increased risk of POAF (adjusted odds ratio ~ 3.2) [18]. These cells exert deleterious effects on atrial myocardium via secretion of pro-inflammatory cytokines (e.g., TNF-α, IFN-γ), promotion of oxidative stress, and direct cytotoxic injury, which together disturb ionic channel expression, gap junction integrity, and provoke electrical remodeling. T-cell infiltration also contributes to systemic blood pressure elevation through multiple mechanisms, including cytokine-mediated endothelial dysfunction, increased vascular oxidative stress, and activation of the RAAS, which promote vasoconstriction and sodium retention [16, 19, 20]. Paradoxically, T-cells can also directly modulate vascular tone via the release of vasoactive mediators such as interleukin-17 and nitric oxide, producing localized vasodilatory effects in certain vascular beds [21]. Additionally, T-cell subsets, including Th17/Treg imbalance, promote atrial tissue remodeling, fibrosis, and structural heterogeneity, facilitating reentry circuits and prolonging conduction times. Emerging evidence indicates that T-cell infiltration in the left atrial appendage increases progressively from sinus rhythm to paroxysmal and ultimately persistent atrial fibrillation. Specifically, the density of CD3⁺ T-cells rises in parallel with the severity of atrial remodeling, suggesting that T-cell accumulation not only contributes to POAF initiation but also sustains the structural and electrophysiological substrate necessary for its maintenance [19, 20]. Together, these immunologic alterations reduce atrial effective refractory period dispersion, increase conduction heterogeneity, and heighten atrial susceptibility to triggers such as transient ischemia, autonomic shifts, or perioperative stress, thereby facilitating the onset of POAF.

In the perioperative setting, NSAIDs are frequently administered in combination with glucocorticoids such as dexamethasone as part of multimodal analgesic regimens [12]. Because glucocorticoids also inhibit prostaglandin pathways, their concurrent use with NSAIDs can result in excessive suppression of protective prostaglandin signaling. This exaggerated inhibition promotes fluid retention and hemodynamic stress, thereby increasing atrial wall tension and enhancing the substrate for atrial electrical instability, ultimately facilitating the development of POAF.

**Fig. 2:**
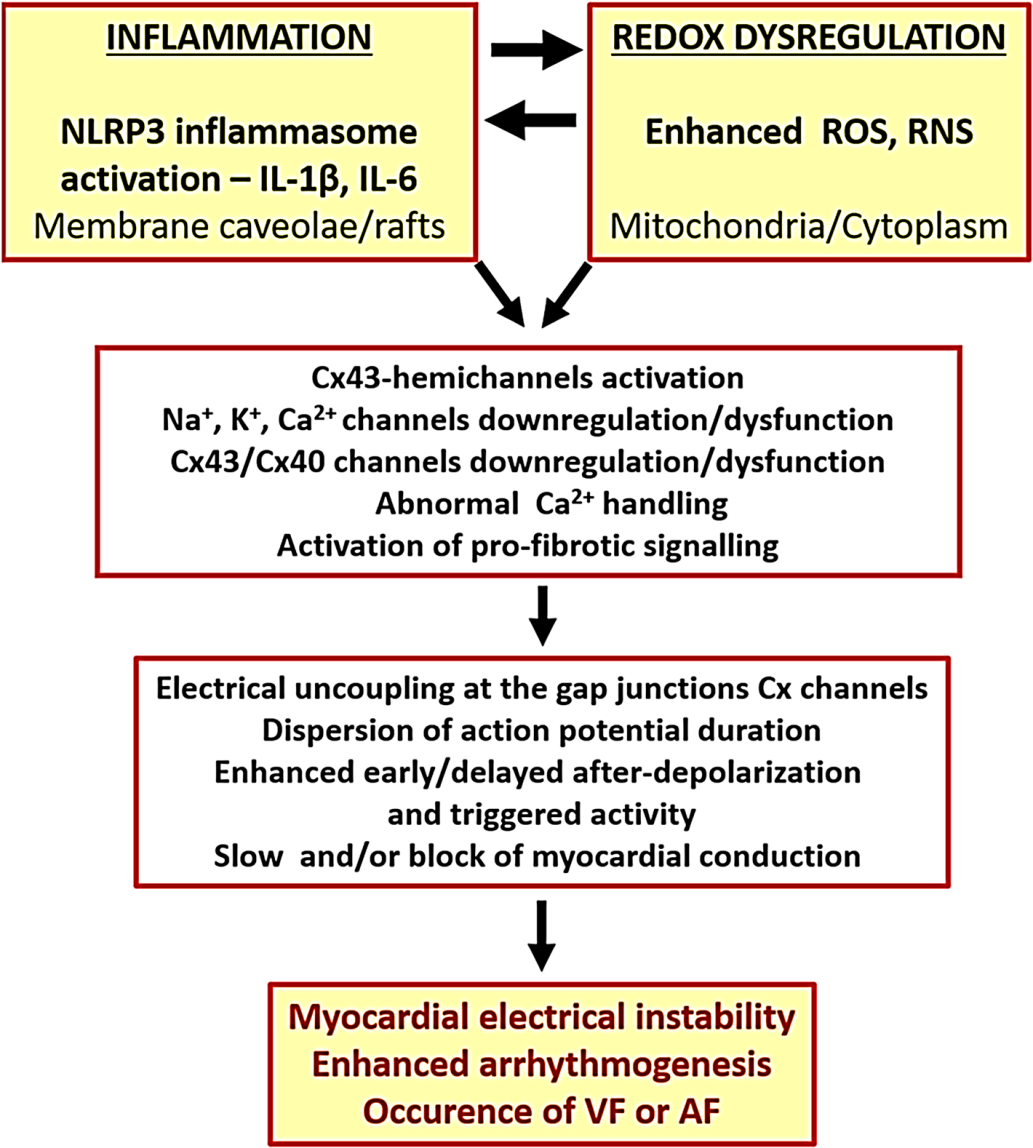
Proposed mechanisms linking cardiac arrhythmias to inflammation and oxidative stress. Diverse external and internal stressors promote production of ROS and activation of the NLRP3 inflammasome, which recruits caspase-1 to process pro–interleukin-1 beta (IL-1β) and pro–interleukin-18 (IL-18) into their active cytokine forms. Resulting inflammatory mediators and redox imbalance drive electrical and structural remodeling of the myocardium — including ion-channel dysfunction, gap-junction (connexin) alterations, fibrosis, and conduction heterogeneity — that create a substrate for arrhythmia initiation and maintenance. Arrhythmic episodes further amplify oxidative stress and inflammatory signaling, establishing a self-perpetuating cycle that contributes to disease progression. NLRP3, NOD-like receptor family pyrin domain containing 3; IL-1β, interleukin-1 beta; IL-6, interleukin-6; ROS, reactive oxygen species; RNS, reactive nitrogen species; VF, ventricular fibrillation; AF, atrial fibrillation. Adapted from reference [25]

Beyond COX inhibition, NSAIDs exert COX-independent effects that directly promote oxidative stress and alter endothelial function. While some NSAIDs have demonstrated antioxidant properties, they can also induce reactive oxygen species generation through interactions with enzymes like NADPH oxidase, xanthine oxidase, and lipoxygenase, contributing to a complex relationship between NSAID use, oxidative stress, and endothelial health [22–24]. When these effects are combined with overlapping COX-inhibitory actions of glucocorticoids, the result is a myocardial environment characterized by local inflammation, cytokine release, oxidative injury, and impaired conduction. These changes foster both structural and electrophysiological remodeling of atrial tissue, creating a pro-arrhythmic substrate (Fig. 2) [25].

Taken together, NSAID administration may paradoxically elevate the risk of POAF through three interrelated mechanisms: (1) disruption of the RAAS, resulting in atrial stretch, (2) additive suppression of prostaglandin pathways when co-administered with glucocorticoids, and (3) COX-independent promotion of oxidative and inflammatory stress. Collectively, these processes contribute to the so-called “NSAID paradox,” wherein agents designed to attenuate inflammation may inadvertently increase atrial susceptibility to postoperative arrhythmias.

### Platelet aggregation

Inhibition of COX-2 can disrupt the delicate balance between pro- and anti-thrombotic factors, potentially enhancing platelet aggregation and increasing the risk of clot formation and atrial remodeling. Patients experiencing moderate to severe intraoperative hemorrhage, shock, or major hemodynamic instability are particularly susceptible to these effects, as platelet hyperresponsiveness in these settings elevates the risk of microthrombotic or thrombotic events [26]. In addition, through the release of immunomodulatory chemicals and indirect modulation of innate leukocyte activity [27], platelets drive a sterile inflammatory response to surgical injury. Beyond their hemostatic role, platelets are critical mediators of vascular repair, angiogenesis, and immune defense against infection [28]. However, platelet activation may amplify inflammatory cascades by releasing cytokines, chemokines, and growth factors that recruit leukocytes and sustain local tissue injury [29–31]. Activated platelets also interact with endothelial cells, inducing endothelial dysfunction and oxidative stress [32]. Collectively, these processes promote atrial structural and electrical remodeling, thereby creating a substrate conductive to the development of POAF.

### Autonomic nervous system dysregulation

Nonsteroidal anti-inflammatory drugs have been postulated to influence autonomic regulation by perturbing the equilibrium between the sympathetic and parasympathetic nervous systems, thereby fostering a milieu that may predispose to POAF [33]. This autonomic imbalance is thought to be primarily mediated through suppression of prostaglandin synthesis, which modulates vascular tone and baroreceptor sensitivity. By attenuating baroreflex function, NSAIDs may shift the autonomic profile toward sympathetic predominance, an effect reported to be more pronounced with COX-2 selective agents [34, 35]. Disruption of this balance has been mechanistically linked to enhanced atrial excitability and an increased propensity for arrhythmogenesis [36]. Importantly, not all NSAIDs appear to exert an equal impact on autonomic regulation; pharmacodynamic heterogeneity across the class suggests that specific agents may confer greater autonomic perturbation than others [37, 38]. There is some evidence to suggest that these autonomic effects may be dose-dependent, with higher cumulative or peak exposures associated with greater shifts in autonomic tone [39, 40]. Furthermore, NSAID-induced alterations in autonomic regulation may lead to renal impairment, which in turn heightens the risk of arrhythmia. By reducing renal perfusion and impairing sodium and potassium homeostasis, NSAIDs can precipitate electrolyte disturbances — particularly hypokalemia and hypernatremia — that further increase susceptibility to atrial arrhythmias. While the biological plausibility of NSAID-induced autonomic and renal-mediated mechanisms is recognized, the clinical relevance remains uncertain. Consequently, further investigation is warranted to delineate the extent of dose dependency, the variability across individual NSAIDs, and the actual magnitude of clinical risk attributable to these mechanisms.

### Electrolyte imbalance and underlying cardiovascular conditions

Nonsteroidal anti-inflammatory drugs can impair renal function, potentially leading to clinically significant electrolyte disturbances and cardiac arrhythmias. Furthermore, patients undergoing surgery frequently exhibit pre-existing cardiovascular comorbidities that may be exacerbated by NSAID-induced renal and electrolyte abnormalities, thereby increasing the risk of POAF. Importantly, even in the absence of other nephrotoxic agents, NSAIDs alone have been shown to induce acute kidney injury, disrupt fluid and electrolyte balance, and promote volume overload. Although aminoglycosides are not routinely administered in the perioperative setting, their concomitant use with NSAIDs — when indicated — may result in synergistic nephrotoxic effects. Such combined exposures may worsen renal impairment, aggravating hypervolemia and venous congestion, impairing tissue perfusion and cellular oxygenation, and triggering activation of the sympathetic nervous system and the RAAS [41]. These hemodynamic and neurohormonal alterations may compromise myocardial electrophysiology, thereby lowering the threshold for POAF development.

## Evidence from translational and clinical studies

### Translational evidence

Pulmonary veins (PVs) are recognized as key anatomical and electrophysiological triggers of atrial fibrillation, with ectopic activity originating from the PVs often initiating and sustaining arrhythmic episodes. Experimental evidence from rabbit models has demonstrated that both selective and non-selective NSAIDs, at clinically relevant concentrations, can alter the electrical properties of the PVs and atrial myocardium. Specifically, selective inhibition of COX-2 by celecoxib was found to induce delayed afterdepolarizations and burst firing specifically within PV tissue [42] — key electrophysiological phenomena implicated in arrhythmia initiation.

Although NSAIDs generally do not affect the resting membrane potential, celecoxib has been shown to significantly shorten atrial action potential duration and reduce sinoatrial node firing rates [41, 42]. In contrast, non-selective inhibition of both COX-1 and COX-2 do not produce notable changes in right atrial action potential duration or spontaneous activity in the PVs or sinoatrial node [42]. Mechanistically, celecoxib enhances the sodium–calcium exchanger current, a proarrhythmic effect that promotes calcium overload and triggered activity in PV cardiomyocytes [43]. These findings suggest a potential mechanistic link between selective COX-2 inhibition and POAF pathogenesis via direct modulation of PV electrophysiology. By increasing ectopic activity within this arrhythmogenic substrate, celecoxib may lower the threshold for POAF onset, particularly in susceptible individuals.

In rodent models, selective ablation of cardiomyocyte COX-2 expression induces interstitial and perivascular fibrosis, predisposing the heart to ischemia-related arrhythmias [43]. Moreover, COX-2 inhibition disrupts PV burst firing and sodium current in ganglionic cells, and alters vascular tone and excitability [43].

Clinically, more than 10% of patients undergoing non-cardiac surgery develop myocardial injury (MINS), a common and prognostically significant condition associated with an increased risk of cardiovascular complications and mortality. Experimental studies in rodents have shown that resident CCR2 − MHCIIlow (or M2) macrophages — embryonically derived and maintained in the adult heart under steady-state conditions — play key roles in supporting tissue repair and anti-inflammatory responses [44–47]. Furthermore, these cells promote electrical activity in the atrioventricular node and inhibit fibrosis [48, 49]. However, following myocardial infarction, this protective macrophage population declines rapidly — by approximately 60% within two days — primarily due to cell death [50, 51]. In the setting of MINS, necrotic cardiomyocytes and macrophages release danger-associated molecular patterns (DAMPs), leading to rapid myocardial swelling and robust activation of the innate immune response. Notably, DAMP binding to Toll-like receptors triggers inflammatory signaling pathways and the release that recruit and activate resident and infiltrating leukocytes to clear necrotic tissue and debris [52–55]. Additionally, depletion of cardiac macrophages contributes to ventricular dysfunction, metabolic dysregulation, inflammasome activation, impaired cardiomyocyte autophagy, accumulation of damaged mitochondria, and defective clearance of exophores (i.e., particles containing dysfunctional mitochondria and other cellular components) [55].

**Fig. 3:**
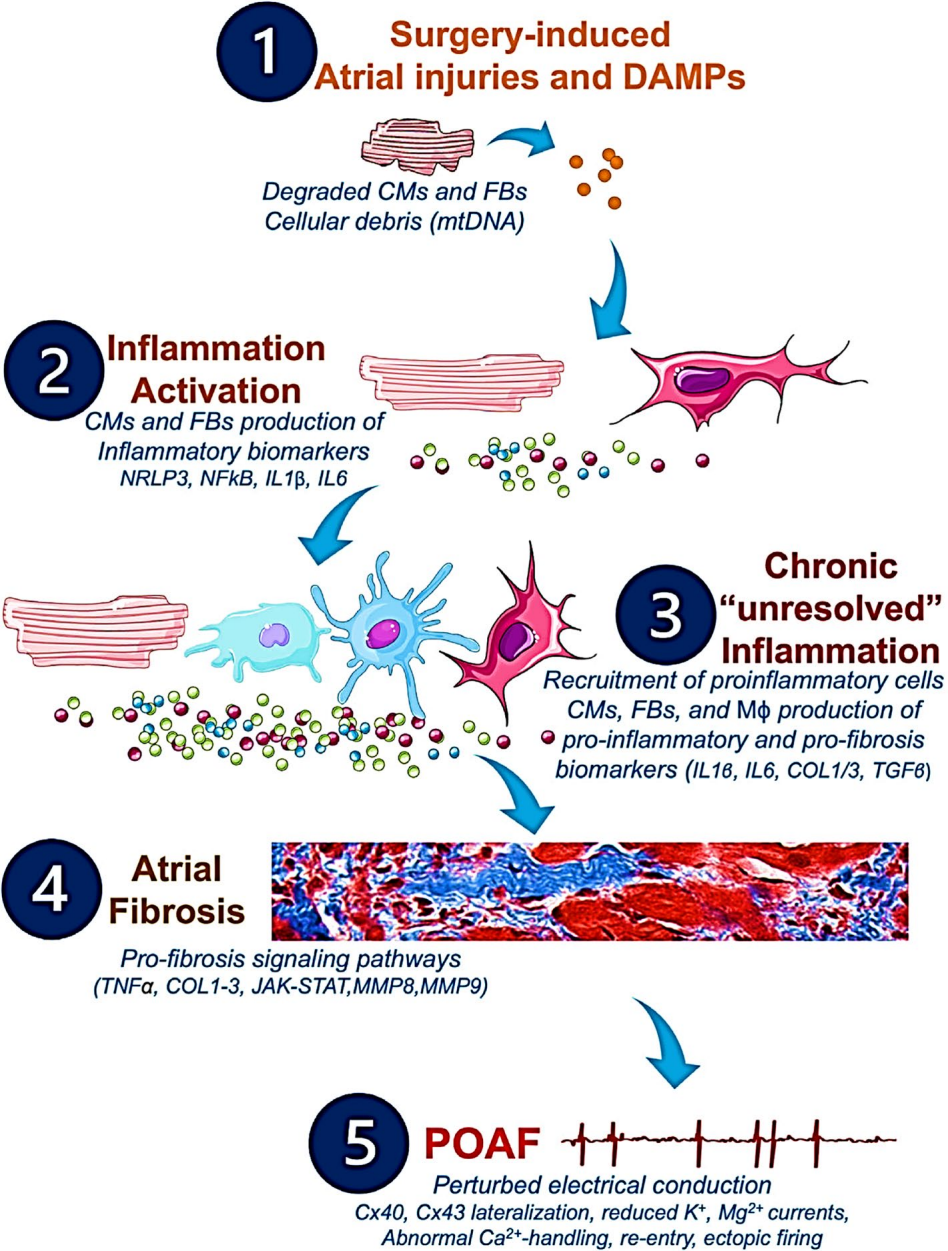
Potential pathophysiological cascade and molecular pathways to be targeted by future anti-inflammatory and anti-fibrosis treatments in POAF management. Cardiac and non-cardiac surgeries may provoke alterations of the CMs and FBs, which activate the inflammatory response, leading to production of further inflammatory biomarkers responsible for the remodeling of the atrial tissue, the development of atrial fibrosis, and the installation of POAF. Ca^2+^, calcium; CMs, cardiomyocytes; COL1, collagen 1; COL3, collagen 3; Cx, connexin; DAMPs, damage-associated molecular patterns; FBs, fibroblasts; IL1β, interleukin 1β; IL6, interleukin 6; JAK/STAT, Janus kinase signal transducer and activator of transcription; K^+^, potassium; Mϕ, macrophages; Mg.^2+^, magnesium; MMPs, matrix metalloproteinases; MnSOD, manganese-dependent superoxide dismutase; mtDNA, mitochondrial DNA; NFkB, nuclear factor kappa B; NLRP3, NOD-like receptor family, pyrin domain containing 3; TGFβ, transforming growth factor beta; TNFα, tumor necrosis factor alpha. Adapted from reference [58]

In patients with heart failure, activation of the left atrial JAK-STAT pathway may occur, enhancing platelet-derived growth factor-signaling [56]. This pathway exerts profibrotic effects, promoting fibroblast activation in vitro and augmenting left atrial fibrosis and remodeling in vivo in post-myocardial infarction mice [56]. Postoperative atrial fibrillation is further facilitated by activation of the NLRP3 inflammasome in macrophages, fibroblasts, and cardiomyocytes [57]. Notably, inhibition of the JAK–STAT pathway reduces fibroblast proliferation and cardiomyocyte hypertrophy in response to pressure overload, underscoring its contribution to atrial structural remodeling and AF susceptibility (Fig. 3) [57, 58]. Inappropriate or excessive perioperative NSAID use may influence these inflammatory and fibrotic mechanisms, thereby increasing the risk of POAF. Table 1 summarizesthe proposed pathways through which NSAIDs may elevate POAF risk [42, 59].

**Table 1:** Potent mechanisms by which NSAIDs increase the risk of POAF

Exert cardiotoxic effects attributed to the cytochrome p450-mediated alteration of arachidonic acid metabolism and reduction of monoepoxides derived from polyunsaturated fatty acids
Induce the formation of reactive oxygen species in different cell types, including cardiac and cardiovascular-related cells, thereby altering key intracellular signaling pathways
Affect left ventricular function, even after a short treatment exposure
Disturb the production of thromboxane and prostacyclin by inhibiting COX-1 and COX-2 enzymes, shifting the prothrombotic/antithrombotic balance on endothelial surfaces toward thrombosis
Suppress pulmonary vein and sinoatrial node spontaneous beating rates, which are known to induce burst firings facilitate, delayed afterdepolarizations, and enhance atrial arrhythmogenesis
Inhibition of COX-2 shortens the action potential duration in both atria, facilitating the maintenance of re-entrant circuits
Shorten the action potential duration only in the left atrium, creating an inter-atrial dispersion and re-entrant circuits
Selective deletion of cardiomyocyte COX-2 expression induces interstitial and perivascular fibrosis, predisposing to ischemic arrhythmias
Inhibition of COX-2 affects sodium current and reduces burst firings in ganglionic cells and pulmonary veins, opens voltage-gated potassium channels and blocks L-type calcium channels, and changes the vascular tone and excitability
May cause acute kidney injury leading to fluid retention, electrolyte disturbances, and hypertension

Data are from experimental and clinical studies

*COX* cyclooxygenase-2

Collectively, these experimental findings offer mechanistic insights directly relevant to the perioperative setting. The observed effects of COX inhibition on atrial electrophysiology, oxidative stress, and inflammatory signaling provide a biologically plausible basis for the clinical associations discussed in the following sections. While animal models cannot fully recapitulate the complexity of human surgical physiology, they elucidate key pathophysiological pathways that may contribute to the heightened susceptibility to POAF observed in clinical populations.

### Clinical evidence

Evidence suggests a potential association between NSAID use and the development of POAF following non-cardiac surgery. This relationship was indicated in a pooled analysis of two randomized double-blind clinical trials and three observational studies. In one trial evaluating POAF incidence among 608 patients (mean age ~48 years; 331 abdominal/pelvic surgeries; 277 orthopedic surgeries) treated with hydroxypropyl-β-cyclodextrin–diclofenac (18.75 mg or 37.5 mg; in orthopedic patients, 18.75 mg, 37.5 mg, or 50 mg depending on predefined risk factors), ketorolac (30 mg), or placebo (mean doses: 226.7 ± 138.7 mg, 206.6 ± 92.1 mg, and 0 mg, respectively), only one patient (0.3%) developed POAF [60]. Adverse events were monitored from baseline through 30 d after initial study drug administration in the abdominal/pelvic surgery cohort and through 30-37 d after the last administration in the orthopedic cohort [60].

In a large population-based study of 1,520,109 patients without a prior history of atrial fibrillation, 6,048 (0.4%) developed in-hospital POAF after receiving an NSAID (ATC code M01A) during non-cardiac surgery, most commonly orthopedic procedures (30.2%) [median age 77 (69–84) years; 43.2% men] [61]. Notably, patients who developed POAF had a significantly higher risk of all-cause mortality within one compared with those who did not (HR 1.83; 95% CI 1.67 to 2.01) [61]. Similarly, in a cohort of 2,048 consecutive elective non-cardiac surgery patients (mean age 72 ± 12 years) without prior atrial fibrillation, 44 (2.1%) developed POAF, with no significant difference in baseline acetylsalicylic acid therapy between those with and without POAF [17 (39%) vs. 697 (35%), p = 0.621] [62]. Furthermore, among 131 thoracic surgery patients (> 60 years) at risk for arrhythmia, NSAID use was associated with a higher incidence of POAF (OR 1.43) [63].

A comprehensive methodological critique of these studies — including consideration of confounding factors such as NSAID dosage, surgical type, and study quality — has been presented in our previously published systematic review [64], to which readers are referred for an in-depth evaluation.

## Non-steroidal anti-inflammatory drugs versus aspirin

A recent systematic review reported that acetylsalicylic acid appears to reduce the incidence of POAF following non-cardiac surgery, although the effect did not reach statistical significance (RR 0.92; 95% CI 0.81 to 1.04); *p* = 0.165) [64]. When acetylsalicylic acid was excluded from the analysis, the use of non-aspirin NSAIDs was associated with a significantly increased risk of POAF (RR 1.15; 95% CI 1.07 to 1.23); *p* < 0.001) [64]. These findings align with an international randomized trial investigating colchicine for the prevention of POAF after thoracic surgery, which similarly reported no increase in POAF incidence among patients receiving aspirin [65]. It remains unclear whether acetylsalicylic acid’s apparent protective effect arises from its intrinsic pharmacologic properties or from concomitant use of other cardioprotective agents [64]. Importantly, the 2014 American College of Cardiology/American Heart Association guidelines emphasize that the occurrence of POAF should prompt evaluation for underlying causes, including potential drug toxicity [66]. Collectively, these observations highlight the potential influence of NSAID class on the development of POAF following non-cardiac surgery.

## Clinical and research implications

Perioperative pain management continues to benefit substantially from the use of NSAIDs. However, growing clinical and translational evidence suggests that NSAID use may be associated with an increased risk of POAF following non-cardiac surgery. Although the current data cannot establish causality, the observed association warrants further investigation. Accordingly, perioperative physicians should carefully balance the analgesic benefits of NSAIDs against their potential proarrhythmic and cardiovascular risks, particularly in patients predisposed to POAF.

Nonsteroidal anti-inflammatory drugs exhibit varying degrees of COX-1 and COX-2 selectivity due to structural differences, and they may also differentially influence the RAAS, although these effects are less well characterized [67–69]. Cyclooxygenase-1–selective agents are primarily responsible for serious gastrointestinal adverse events [70], whereas COX-2 inhibition mediates the analgesic and antihyperalgesic effects of NSAIDs [71]. Conversely, agents with higher COX-2 selectivity have been linked to a dose-dependent increase in cardiovascular events. Importantly, the extent of COX inhibition depends on both drug concentration and dosing regimen, which can alter clinical outcomes and toxicity profiles.

Extended duration of NSAID use, higher doses, and rapid infusion rates are generally associated with an increased incidence of adverse effects, including POAF [72–74]. Observational data — mostly from non-surgical populations — suggest that the risk of NSAID-associated POAF may be heightened in individuals with pre-existing cardiovascular disease, advanced age, or other established arrhythmogenic risk factors [1, 40, 66, 72–74]. Additionally, patients receiving concomitant therapies that modulate autonomic tone or hemodynamic function, such as β-blockers or angiotensin-converting enzyme inhibitors, may exhibit altered responses to NSAID exposure; however, these interactions have not been systematically evaluated in the perioperative setting. Certain non-cardiac surgeries characterized by intense inflammation or fluid shifts may further amplify POAF risk, with NSAIDs potentially exacerbating this susceptibility [75]. Comprehensive preoperative assessment and consideration of alternative pain management modalities, such as regional anesthesia and non-pharmacological strategies, may help mitigate NSAID reliance. Close monitoring and correction of perioperative electrolyte imbalances are also essential in NSAID-treated patients.

Emerging inflammatory biomarkers may provide additional insight into the pathophysiological link between NSAIDs, inflammation, and POAF. Soluble urokinase plasminogen activator receptor (suPAR), a marker of persistent low-grade or chronic inflammation, has been associated with adverse cardiovascular outcomes and lifestyle-related risk factors such as smoking, alcohol use, and physical inactivity. Both experimental and clinical studies suggest that elevated suPAR levels correlate with myocardial strain, cardiac failure, thrombosis, and vascular inflammation [76, 77]. Moreover, suPAR has been identified as a predictor of atrial fibrillation across multiple populations [78–80]. Preoperative suPAR concentrations are associated with impaired microcirculatory flow [7, 81], a phenomenon frequently observed in atrial fibrillation [82, 83], and independently predicts postoperative complications in patients undergoing major non-cardiac surgery [6]. Importanlty, despite activation of the systemic inflammatory response, perioperative suPAR levels appear minimally affected by general anesthesia or surgical trauma [8], supporting its potential utility in patient-specific inflammatory and arrhythmia risk stratification.

Further research is needed to better define the patient populations most vulnerable to NSAID-related POAF and to elucidate the underlying mechanisms driving this association. Advancing the understanding of how NSAIDs influence atrial electrophysiology and inflammation will be crucial to developing safer and more effective postoperative pain management strategies. Future research priorities include:

- **Randomized controlled trials:** Well-designed studies are required to determine causality between NSAID use and POAF after non-cardiac surgery, addressing the limitations and bias inherent in existing observational data [12].
- **Risk stratification:** Development of predictive tools to identify patients at high risk for NSAID-induced POAF.
- **Alternative analgesic strategies:** Evaluation of multimodal and non-NSAID-based pain management approaches to reduce NSAID dependence.
- **Mechanistic studies:** Integrated translational research to clarify the molecular and inflammatory pathways by which NSAIDs contribute to POAF and to inform targeted preventive interventions.

## Conclusions

Accumulating translational and clinical evidence supports the biological plausibility that NSAIDs may contribute to the development of POAF following non-cardiac surgery. Mechanistic pathways — including COX inhibition, RAAS activation, oxidative stress, and autonomic dysregulation — offer a coherent framework for this association. However, because the current evidence is largely derived from observational studies characterized by confounding factors, heterogeneous surgical populations, and limited dose–response data, definitive causal inference remains uncertain. Given that NSAID-based OFA strategies have not demonstrated consistent early analgesic superiority over opioid-containing regimens, and may introduce additional complications, perioperative physicians should exercise cautious, individualized decision-making until adequately powered randomized controlled trials clarify the magnitude of risk and identify vulnerable subgroups. This approach includes prudent patient selection, avoidance of unnecessarily high NSAID doses or prolonged use, vigilant monitoring of fluid and electrolyte balance, and the consideration of non-NSAID alternatives when appropriate. Integrating these principles into enhanced recovery after surgery (ERAS) pathways, with active participation from perioperative pharmacists and multidisciplinary teams, may optimize pain management while mitigating inflammation-driven arrhythmic risk.

## Data Availability

No datasets were generated or analysed during the current study.

## Abbreviations

POAF: Postoperative atrial fibrillation
NSAIDs: Non-steroidal anti-inflammatory drugs
COX: Cyclooxygenase
RAAS: Renin-angiotensin-aldosterone system
MINS: Myocardial injury after noncardiac surgery
DAMPs: Danger-associated molecular patterns
